# A case-control study of lower urinary-tract infections, associated antibiotics and the risk of developing prostate cancer using PCBaSe 3.0

**DOI:** 10.1371/journal.pone.0195690

**Published:** 2018-04-12

**Authors:** Beth Russell, Hans Garmo, Kerri Beckmann, Pär Stattin, Jan Adolfsson, Mieke Van Hemelrijck

**Affiliations:** 1 King’s College London, Division of Cancer Studies, Translational Oncology & Urology Research (TOUR), London, United Kingdom; 2 Regional Cancer Centre, Uppsala, Sweden; 3 University of South Australia, Centre for Population Health Research, Adelaide, Australia; 4 Department Surgical Sciences, Uppsala University, Uppsala, Sweden; 5 CLINTEC department, Karolinska Institutet, Stockholm, Sweden; University of South Alabama Mitchell Cancer Institute, UNITED STATES

## Abstract

**Objectives:**

To investigate the association between lower urinary-tract infections, their associated antibiotics and the subsequent risk of developing PCa.

**Subjects/Patients (or materials) and methods:**

Using data from the Swedish PCBaSe 3.0, we performed a matched case-control study (8762 cases and 43806 controls). Conditional logistic regression analysis was used to assess the association between lower urinary-tract infections, related antibiotics and PCa, whilst adjusting for civil status, education, Charlson Comorbidity Index and time between lower urinary-tract infection and PCa diagnosis.

**Results:**

It was found that lower urinary-tract infections did not affect PCa risk, however, having a lower urinary-tract infection or a first antibiotic prescription 6–12 months before PCa were both associated with an increased risk of PCa (OR: 1.50, 95% CI: 1.23–1.82 and 1.96, 1.71–2.25, respectively), as compared to men without lower urinary-tract infections. Compared to men with no prescriptions for antibiotics, men who were prescribed ≥10 antibiotics, were 15% less likely to develop PCa (OR: 0.85, 95% CI: 0.78–0.91).

**Conclusion:**

PCa was not found to be associated with diagnosis of a urinary-tract infection or frequency, but was positively associated with short time since diagnoses of lower urinary-tract infection or receiving prescriptions for antibiotics. These observations can likely be explained by detection bias, which highlights the importance of data on the diagnostic work-up when studying potential risk factors for PCa.

## Introduction

Inflammation caused by infectious agents or environmental conditions has now been put forward as a possible cause for an increased prostate cancer (PCa) risk. Lower urinary tract infections are one example of such infections that are known to cause inflammation [1]. In addition, certain microorganisms have been implicated in the role of carcinogenesis [2]. The microorganism *Escherichia coli* has been identified as being the major pathogen involved in lower urinary-tract infection and is believed to account for around 80% of all urinary tract infections [3,4]. It has however not yet been proven to be carcinogenic.

Inflammation of the prostate gland (prostatitis) is also known to be attributed by infection of the microorganisms that are causative of lower urinary tract infections (LUTIs). With a link between the infectious agents, inflammation and the location of the prostate gland, a potential association between lower urinary-tract infections and PCa may be conceived [5].

Further links between infections and PCa are found with the gram-positive bacillus, *Propionibacterium acnes* as well as the most common non-viral sexually transmitted infection *Trichomonas vaginalis*. It has been proposed that the inflammatory response and prostatitis that occurs from these pathogens may play an important role in the development of PCa [6–8]. Conversely, sexually transmitted infections caused by pathogens such as *Chlamydia trachomatis* and human papillomavirus (HPV) have been found to have little or no association with PCa risk [9,10].

The few studies on the link between lower urinary-tract infections and PCa are inconclusive. One study by Pelucchi and colleagues found that having cystitis increased the men’s risk of having PCa by 76% [11]. On the contrary, a similar study which focused on urethritis, concluded that this particular lower urinary-tract infection was not associated with PCa risk [12].

Using data from the Prostate Cancer Database Sweden (PCBaSe) on diagnosis and prescriptions of antibiotics for lower urinary-tract infections, the current study aims to further disentangle their potential role in the development of PCa.

## Methods

### Data source

All data was obtained from PCBaSe Sweden 3.0 in which data from >98% of PCa cases from the National Prostate Cancer Register (NPCR) of Sweden are recorded [13,14]. This database started in 1998 and encompasses data from around 11 national registers such as the Prescribed Drug Register and the National Patient Register up until 2012. It is comprised of information from 137985 registered PCa men including information based on their records of various demographic and clinical aspects as well as the men’s socio-economic status.

### Study population and study design

From PCBaSe 3.0, we identified 8,762 men who were diagnosed with PCa in 2012 (i.e. cases) and matched them with five controls based on age and county (n = 43,806). Within this cohort, we then identified those who had a diagnosis of a lower urinary-tract infection in the patient register, plus those who had received a lower urinary-tract infection-specific antibiotic according to the National Drug Registry. Matching on county was ignored for those over the age of 90, due to a smaller sample size.

The lower urinary-tract infection diagnoses were defined according to their ICD-10 codes. N30 referred to all diagnoses of cystitis; N34 to urethritis; and N39 to disorder of urinary system caused by infection with unspecified location. For each lower urinary-tract infection diagnosis, the time between first diagnosis and date of being diagnosed with PCa or becoming a control (i.e. date that corresponding case was diagnosed with PCa) was calculated and categorized as 6–12 months, 2–4 years, 5–9 years and ≥10 years. Those lower urinary-tract infections diagnosed within six months prior to PCa diagnosis were not incorporated into the above-described exposure variables, as to avoid reverse causation. For those men who had more than one lower urinary-tract infection, a minimum of 30 days between diagnoses was required.

A total of nine antibiotics were included in the study, all of which have been indicated to be used for lower urinary-tract infections specifically: Trimethoprim/sulfamethoxazole (ATC: J01XX01), Trimethoprim (J01EA01), Ciprofloxacin (J01MA02), Levofloxacin (J01MA12), Cephalexin (J01DB01), Doxycycline (J01AA02), Nitrofurantoin (J01XE01), Amoxicillin/Clavulanate (J01CR02) and Norfloxacin (J01MA06). The number of antibiotic prescriptions, as well as the time between first prescription and event, were also calculated and categorized as: 6–12 months, 1–2 years, 3–4 years and ≥5 years. Prescriptions within 6 months of diagnosis of prostate cancer were not considered as exposed, again to avoid reverse causation. For those men who had more than one prescription of antibiotics, a minimum of 7 days between prescriptions was required. The new prescription could have been for either a different antibiotic or the same.

Furthermore, we collected information on Charlson Comorbidity Index (CCI). The latter assigns weights to a number of medical conditions, including diabetes and hypertension, based on discharge diagnoses prior to the date of diagnosis in the Patient Register [15]. Education was divided into three categories; Low referred to those who spent ≤10 years in the Swedish education system; Medium included the men with 10–12 years’ experience in Swedish schooling; High included those who went to university.

### Statistical analysis

Odds ratios (ORs) were calculated using conditional multivariate logistic regression models. The various exposure variables describing the diagnosis of lower urinary-tract infections or use of antibiotics were studied in relation to risk of being diagnosed with PCa. All ORs were adjusted for civil status, education and CCI. The ORs, with respect to lower urinary-tract infections, were additionally adjusted for ‘time between first lower urinary-tract infection diagnosis and event’, whilst the ORs for exposure variables solely relating to antibiotics were instead additionally adjusted for the ‘time between first antibiotic and event’.

All data management was performed on SAS version 9.3 (SAS Institute, Cary, NC, USA), whilst all statistical analysis was performed on STATA/IC 12.1 (Texas, USA).

## Results

Cases and controls showed similar distributions for age, education level, civil status and CCI (Table 1). Table 2 displays the PCa specific variables for the cases. In total, 9% of men were found to have distant metastatic PCa, while 3% of cases were recorded as having metastasis to their lymph nodes local to the prostate. The mean PSA at time of diagnosis for all men with PCa was 8.6 μg/ml.

**Table 1:** Characteristics of cases and controls selected from PCBaSe 3.0.

Variable	Cases n (%)	Controls n (%)
**Age**		
40–49	81 (0.92)	431 (0.98)
50–59	1072 (12.23)	5341 (12.19)
60–69	3577 (40.82)	17816 (40.67)
70–79	2785 (31.78)	13997 (31.95)
80+	1247 (14.23)	6221 (14.20)
Mean (SD)	69.84 (8.98)	69.83 (8.99)
**Educational Level**		
High	2254 (25.72)	10374 (23.68)
Low	2956 (33.74)	15535 (35.46)
Middle	3491 (39.84)	17285 (39.46)
Missing	61 (0.70)	612 (1.40)
**Civil Status**		
Married	5832 (66.56)	27201 (62.09)
Not married	1052 (12.00)	6140 (14.02)
Divorced	1280 (14.61)	5832 (13.31)
Widower	596 (6.80)	3258 (7.44)
Missing data	2 (0.02)	52 (0.12)
**CCI**		
0	6062 (69.19)	29226 (66.72)
1	1304 (14.88)	6887 (15.72)
2	817 (9.32)	4101 (9.36)
3+	579 (6.61)	3592 (8.20)

CCI = Charlson comorbidity index

**Table 2:** PCa specific characteristics of cases selected from PCBaSe 3.0.

Variable	Cases n (%)	Controls n (%)
**Gleason Score**		
<7	3263 (37.24)	-
7	3285 (37.49)	-
>7	1982 (22.62)	-
Missing data	232 (2.65)	-
**T Stage**		
T0	31 (0.35)	-
T1a	263 (3.00)	-
T1b	157 (1.79)	-
T1c	4083 (46.60)	-
T2	2421 (27.63)	-
T3	1350 (15.41)	-
T4	280 (3.20)	-
TX	171 (1.95)	-
Missing data	6 (0.07)	-
**N-Stage**		
N0	1960 (22.37)	-
N1	220 (2.51)	-
NX	6567 (74.95)	-
Missing data	15 (0.17)	-
**M-Stage**		
M0	7957 (90.81)	-
M1	788 (8.99)	-
MX	2 (0.02)	-
Missing data	15 (0.17)	-
**Number of biopsy cores taken**		
<10	2265 (25.85)	-
10–19	5723 (65.32)	-
20–29	45 (0.51)	-
30+	4 (0.05)	-
None	725 (8.27)	-
**Number of biopsy cores with cancer**		
0	1 (0.01)	-
1–4	5012 (57.20)	-
5–9	2313 (26.40)	-
10–14	605 (6.90)	-
15+	1 (0.01)	-
None	830 (9.47)	-
**PSA at time of diagnosis (μg/ml)**		
Median (IQR)	8.6 (15.7)	-

PSA = prostate specific antigen.

The frequency, type, and time since lower urinary-tract infection diagnoses are displayed in Table 3. The frequency of lower urinary-tract infections was similar across both cases and controls, with 5% of cases and controls having had 1–2. More men were diagnosed with a lower urinary-tract infection of unspecified location than any other type (4% of cases and controls). The adjusted ORs displayed in Table 3 only indicate a statistically significant association with PCa for the period of time between initial lower urinary-tract infection and PCa diagnosis: OR for lower urinary-tract infection 6–12 months prior to PCa diagnosis: 1.50 (95% CI: 1.23–1.82).

**Table 3:** Odds ratios (OR) and 95% confidence intervals (CI) for PCa based on history of lower urinary-tract infection.

	Cases N (%)	Controls N (%)	Crude 0R	95% CI	Adjusted OR	95% CI
**Number of lower urinary tract infections**						
0	8288 (94.59)	41457 (94.64)	1.00	Ref.	1.00^1^	Ref.
1–2	454 (5.18)	2224 (5.08)	1.02	(0.92–1.13)	0.96	(0.72–1.30)
3–4	15 (0.17)	102 (0.23)	0.74	(0.43–1.27)	0.75	(0.40–1.40)
5+	5 (0.06)	23 (0.05)	1.09	(0.41–2.86)	1.13	(0.41–3.11)
**Type of lower urinary-tract infections**						
No LUTI	8288 (94.59)	41457 (94.64)	1.00	Ref.	1.00^1^	Ref.
Any	474 (5.41)	2349 (5.36)	1.01	(0.91–1.12)	0.96	(0.71–1.28)
Cystitis N30	77 (0.88)	408 (0.93)	0.94	(0.74–1.21)	0.91	(0.64–1.31)
Urethritis N34	10 (0.11)	54 (0.12)	0.93	(0.47–1.82)	0.90	(0.43–1.87)
LUTI of unspecified location N39	356 (4.06)	1694 (3.87)	1.05	(0.93–1.18)	1.00	(0.73–1.36)
Multiple types	31 (0.35)	193 (0.44)	0.8	(0.55–1.18)	0.84	(0.53–1.33)
**Time between first lower urinary-tract infections diagnosis and event (yrs)**						
No LUTI diagnosed	8288 (94.59)	41457 (94.64)	1.00	Ref.	1.00^2^	Ref.
6–12 months	265 (3.02)	1229 (2.81)	1.41	(1.16–1.70)	1.50	(1.23–1.82)
2–4	156 (1.78)	827 (1.89)	0.87	(0.72–1.05)	0.93	(0.77–1.12)
5–9	53 (0.60)	282 (0.64)	0.94	(0.79–1.22)	0.97	(0.81–1.16)
10+	0 (0)	11 (0.03)	0.91	(0.67–1.21)	0.96	(0.71–1.28)

^1^Adjusted for civil status, education, CCI and time between first UTI diagnosis and event.

^2^Adjusted for civil status, education and CCI.

Date for controls in ‘Time between first LUTI diagnosis and event’ have been taken as the date of the man becoming a control. Multiple types of UTI means any combination of the three types of UTI.

Table 4 shows the distribution of specific antibiotic use among cases and controls, and their corresponding crude and adjusted ORs. Of the men who had been prescribed antibiotics, most had received between 1 and 3 prescriptions (34% cases and 30% controls), whilst considerably fewer men received over 10 (1% cases and controls). The multivariate logistic regression analysis revealed that men who had received any antibiotic were at a 19% increased risk of PCa (OR: 1.19, 95% CI: 1.12–1.27). In addition, trimethoprim/sulfamethoxazole, ciprofloxacin, doxycycline and norfloxacin were all associated with an increased risk of PCa (OR: 1.33, 95% CI: 1.10–1.62; OR: 1.39, 95% CI: 1.28–1.52; OR = 1.10, 95% CI: 1.02–1.18 and OR: 1.25, 95% CI: 1.08–1.46 respectively). Conversely, men who had 10 or more antibiotic prescriptions were 15% less likely to develop PCa (OR: 0.85, 95% CI: 0.79–0.91). With respect to time between the first antibiotic prescription and event, it was found that the majority of cases and controls (60% and 64% respectively), had at least 5 years between these two dates. Time since antibiotic prescription was positively associated with PCa diagnosis, but the strongest association was seen for those men who had 6–12 months between the first antibiotic and PCa diagnosis (OR: 1.96, 95% CI: 1.71–2.25), as compared to men with no prescriptions.

**Table 4:** Odds ratios (OR) and 95% confidence intervals (CI) for PCa based on prescriptions of lower urinary-tract infection-related antibiotics.

	Cases N (%)	Controls N (%)	Crude 0R	95% CI	Adjusted OR	95% CI
**Number of antibiotic prescriptions**						
None	5237 (59.77)	28199 (64.37)	1.00	Ref.	1.00^1^	Ref.
1–3	2961 (33.79)	13030 (29.74)	0.96	(0.85–1.08)	1.02	(0.90–1.15)
4–6	376 (4.29)	1736 (3.96)	1.01	(0.82–1.26)	1.06	(0.85–1.33)
7–9	106 (1.21)	462 (1.05)	0.95	(0.75–1.21)	1.05	(0.82–1.34)
10+	82 (0.94)	379 (0.87)	0.82	(0.76–0.86)	0.85	(0.79–0.91)
**Antibiotic received**						
None			1.00	Ref.	1.00^1^	Ref.
Trimethoprim/sulfamethoxazole	309 (3.53)	1398 (3.19)	1.31	(1.09–1.59)	1.33	(1.10–1.62)
Trimethoprim	313 (3.57)	1475 (3.37)	1.07	(0.89–1.29)	1.10	(0.91–1.33)
Ciprofloxacin	1785 (20.37)	6905 (15.76)	1.41	(1.31–1.51)	1.39	(1.28–1.52)
Levofloxacin	13 (0.15)	65 (0.15)	0.64	(0.25–1.63)	0.65	(0.26–1.66)
Cephalexin	11 (0.13)	31 (0.07)	1.43	(0.53–3.82)	1.49	(0.55–4.00)
Doxycycline	1949 (22.24)	9175 (20.94)	1.12	(1.05–1.19)	1.10	(1.02–1.18)
Nitrofurantoin	156 (1.78)	749 (1.71)	1.14	(0.81–1.61)	1.12	(0.78–1.60)
Amoxicillin & Clavulanate	185 (2.11)	799 (1.82)	1.13	(0.91–1.41)	1.12	(0.89–1.40)
Norfloxacin	314 (3.58)	1348 (3.08)	1.26	(1.09–1.46)	1.25	(1.08–1.46)
Any	-	-	1.22	(1.16–1.28)	1.19	(1.12–1.27)
**Time between first antibiotic and event (yrs)**						
No antibiotic given	291 (3.32)	812 (1.85)	1.00	Ref.	1.00^2^	Ref.
6–12 months	780 (8.90)	3473 (7.93)	1.93	(1.68–2.22)	1.96	(1.71–2.25)
1–2	949 (10.83)	4426 (10.10)	1.21	(1.12–1.32)	1.23	(1.13–1.33)
3–4	1505 (17.18)	6896 (15.74)	1.16	(1.07–1.25)	1.17	(1.08–1.26)
5+	5237 (59.77)	28199 (64.37)	1.18	(1.11–1.26)	1.20	(1.12–1.27)

^1^Adjusted for civil status, education, CCI and time between first antibiotic and event.

^2^Adjusted for civil status, education and CCI.

Type of antibiotic is the first antibiotic received if more than one has been prescribed. Date for controls in ‘Time between first antibiotic and event have been taken as the date of the man becoming a control i.e. date of event for corresponding case.

Finally, results were stratified by PCa risk categories and comparable ORs were produced between low risk and high risk groups for time since UTI diagnosis and antibiotic prescription.

## Discussion

This population-based case-control study did not find any association between lower urinary-tract infections and their frequency with PCa risk. Only those who had a lower urinary-tract infection within the past year were found to have an increased risk of PCa diagnosis. Taking lower urinary-tract infection related antibiotics and the most recent prescriptions or diagnoses were found to be linked with increased odds of PCa diagnosis. However, it needs to be noted that men being prescribed many antibiotics (≥10) were discovered to have a lower risk of PCa.

Our null results for lower urinary-tract infections in relation to PCa risk were somewhat unexpected and are inconsistent with the results reported by Fan et al who found a possible etiological role for urethritis and cystitis in the development of PCa [5]. In particular, they found that urethritis had the most significant increase in PCa risk with an OR of 1.72 (95% CI: 1.26–2.34). This study however, only looked at a cohort of Taiwanese men and hence the findings may not be generalizable to a European population. Conversely, the null finding for lower urinary-tract infections in our study was also established in a similar case control study in which UTIs were studied in relation to bladder cancer [2]. Initially, this investigation looked at all diagnosis of cystitis in relation to bladder cancer and found an OR of 1.52 (95% CI: 1.12–2.06). However, upon excluding any cases of cystitis that occurred within one year prior to diagnosis of bladder cancer, the association disappeared.

In a paper by Severi et al, they reported that an increase in antibodies against the pathogen *P*.*acnes* provided protection against PCa [16]. Therefore, our result that an increased frequency of lower urinary-tract infections did not increase the OR for risk in this present study was surprising. Nonetheless, the majority of lower urinary-tract infections are caused by the pathogen *E*.*coli* therefore these antibodies may not provide the same carcinogenic protection as those of *P*.*acnes* [17].

Our observation that having an lower urinary-tract infection 6–12 months before PCa diagnosis increased the risk of PCa by 49% is somewhat in line with a study by Pelucchi et al [11], who found an OR of 1.76 when the lower urinary-tract infection occurred less than 5 years before PCa diagnosis. These observations are likely to be explained by detection bias as we did not see any association with frequency or type of lower urinary-tract infection. Men who have lower urinary-tract infections are likely to undergo a work-up and hence have an increased risk of detecting PCa. Furthermore, lower urinary-tract infections can also be caused by a bladder outlet obstruction (BOO) as a result of benign prostate hyperplasia (BPH). The lower urinary tract symptoms (LUTS) caused by BPH may then result in a visit to the doctors and thus increased risk of PCa diagnosis [18]. Our observations for time since receiving antibiotics showed a similar trend.

Men who had received any of the lower urinary-tract infection associated antibiotics were at a 19% increased risk of PCa diagnosis. Interestingly, however, men who received 10 or more prescriptions for antibiotics had a 15% lowered risk of developing PCa. No other study to date has researched the use of lower urinary-tract infection-specific antibiotics and PCa, therefore these conflicting results cannot be compared with previous data. However, it can be hypothesized that detection bias is also an issue here. Those who received antibiotics would have been to see a medical practitioner and are subsequently more likely to have any PCa detected. Conversely, the decrease in risk for men who received 10 of more antibiotics may be explained by a decrease in inflammation. One study revealed a chemopreventive role of the antibiotic erythromycin in mice [19]. Hamoya et al hypothesised that the decrease in cancer development was in part caused by the attenuation of local inflammation [19]. However, in our study, it is not possible to tease out this potential chemopreventive role and one could also hypothesize that this decrease in PCa risk is simply due to the fact that these men are longitudinally followed and truly have no PCa.

Strengths of this study include its large sample size, adjustment for a variety of confounders including CCI, and a combination of data on both lower urinary-tract infection diagnosis and antibiotics. However, many antibiotics are commonly prescribed to treat several types of infections and not just lower urinary-tract infections [20]. Penicillins for example (such as amoxicillin) can be used to treat anything from skin infections to chest infections as well as urinary tract infections [20]. Consequently, some prescriptions included in this present study might have been used to treat other indications than lower urinary-tract infections—however this potential misclassification would have been the same for both cases and controls. Another possible limitation to this study was the identification of men with lower urinary-tract infections. PCBaSe does not have detailed information on indication for diagnosis (e.g. confirmation of culture)–again this misclassification bias would have been applicable to both cases and controls. Furthermore, all cases were selected based on their hospital admission. Therefore, it is reasonable to presume that these cases would have encompassed the more severe lower urinary-tract infection diagnoses and so may not be representative of all lower urinary-tract infections.

### Conclusion

We did not find an increased risk of PCa following the diagnosis of a lower urinary-tract infection. However, shortly after the filling of a prescription for an antibiotic or the diagnosis of a lower urinary-tract infection, there was an increased risk of PCa, which suggests detection bias. This thus highlights the importance of data on the diagnostic work-up when studying potential risk factors for PCa. Furthermore, since our results clearly point towards detection bias rather than causality, we feel that this paper means that the existing literature should be assessed carefully.
